# A Review of Western and Traditional Chinese Medical Approaches to Managing Nonalcoholic Fatty Liver Disease

**DOI:** 10.1155/2016/6491420

**Published:** 2016-10-31

**Authors:** Wei-Fan Hsu, Lee-Yan Sheen, Hung-Jen Lin, Hen-Hong Chang

**Affiliations:** ^1^Division of Hepato-Gastroenterology, Department of Internal Medicine, China Medical University Hospital, Taichung, Taiwan; ^2^Institute of Food Science and Technology, National Taiwan University, Taipei, Taiwan; ^3^School of Post-Baccalaureate Chinese Medicine, College of Chinese Medicine, China Medical University, Taichung, Taiwan; ^4^Department of Chinese Medicine, China Medical University Hospital, Taichung, Taiwan; ^5^Research Center for Chinese Medicine & Acupuncture, China Medical University, Taichung, Taiwan

## Abstract

Nonalcoholic fatty liver disease (NAFLD) is a disease of attention because of increase in prevalence from 20% to 41%. The clinical and pathological conditions in patients with NAFLD range from steatosis alone to nonalcoholic steatohepatitis (NASH) with or without fibrosis to hepatic cancer. In the United States, NAFLD was the second-leading indication for liver transplant between 2004 and 2013. Although imaging studies such as magnetic resonance elastography and the use of diagnostic panels and scoring systems can provide a fairly accurate diagnosis of NAFLD, there are few treatment options for patients with mild to moderate disease other than lifestyle modification. Many of the currently used medical treatments have been shown to cause severe side effects and some have been shown to be associated with increased risk for certain types of cancer. In recent years, a number of traditional Chinese herbal treatments have been examined for their potential uses as treatment for NAFLD. In this review, we provide a general overview of NAFLD and a survey of Western pharmacologic drugs currently used to treat the disease as well as the results of recent studies on the effectiveness of traditional Chinese herbal remedies for managing nonalcoholic fatty liver disease.

## 1. Introduction

Nonalcoholic fatty liver disease (NAFLD) is a disease of attention because of increase in prevalence. According to the American Gastroenterological Association (AGA) and the American Association for the Study of Liver Disease (AASLD), NAFLD is defined as the presence of hepatic steatosis on imaging studies or histologic examinations in patients without etiologies for secondary hepatic fat accumulation, such as a history of significant alcohol consumption, use of corticosteroids or amiodarone, hepatitis C viral (HCV) infection, Wilson's disease, or starvation [1]. The disease mainly affects overweight and obese individuals but is also found in lean people. The clinical and pathological conditions in patients with NAFLD range from steatosis alone to nonalcoholic steatohepatitis (NASH) with or without fibrosis to hepatocellular carcinoma (HCC) [2]. Until recently, nonalcoholic fatty liver (NAFL) and NASH were considered two separate clinical entities; however, recent evidence shows that NAFLD represents a continuum of hepatic injuries, which progress from simple steatosis to NASH [3, 4]. In a systematic review of follow-up biopsies, Raluca et al. reported that more than half of patients (16 out of 25) with untreated NAFL developed NASH after a mean follow-up of 3.7 years [3]. In another systematic review and meta-analysis of studies that mainly used Brunt's pathologic classification [5], Singh et al. found that fibrosis progressed by one stage over 14.3 years in patients with NAFL and by one stage over 7.1 years in patients with NASH [4].

## 2. Prevalence of and Risk Factors Associated with NAFLD

**Figure 1:**
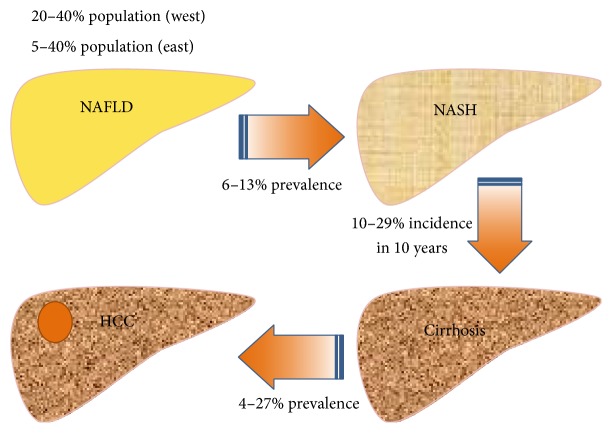
The natural history of NAFLD. The prevalence of NAFLD ranges from 20 to 40% in Western countries and from 5 to 40% in the Asia-Pacific area. As many as 13% (range, 6%–13%) of patients with NAFLD have histological evidence of NASH. Among them, 10% to 29% develop cirrhosis within 10 years and 27% of those patients develop HCC. HCC: hepatocellular carcinoma; NAFLD: nonalcoholic fatty liver disease; NASH: nonalcoholic steatohepatitis.

The prevalence of NAFLD ranges from approximately 20% to 27% in Mainland China and Hong Kong [6] and from 11.4% to 41% in Taiwan [7, 8]. In the United States, the prevalence of NAFLD increased by 170% during the period from 2004 to 2013 and was the second-leading indication for liver transplant during that time period [9]. In Taiwan, about 6% to 13% of patients with NAFLD have histological evidence of NASH. Among them, 10% to 29% develop cirrhosis within 10 years and 4% to 27% of those patients develop HCC (Figure 1) [10].

The increase in prevalence of NAFLD has been attributed to the rise in availability and consumption of foods with high fat, protein, and sugar contents, increasing levels of urbanization and decreasing levels of physical activity [6]. Although the prevalence of NAFLD is higher among people of advanced age, Wong et al. found that the prevalence of the disease was about 30% in individuals aged between 30 and 39 years in Hong Kong [11]. Patients with obesity (body mass index (BMI) ≧ 28), longstanding or persistent abnormalities in aminotransferase levels, older age (>45 years or 50 years in overweight patients), diabetes mellitus, metabolic syndrome, abnormal liver biochemical and function test results, or evidence of fibrosis on an imaging study are more likely to have NASH [2, 12–15]. Liver biopsy is required to establish a definitive diagnosis of NASH; however, given its invasive nature, only patients at high risk for NASH or advanced fibrosis, such as patients with an AST/ALT ratio greater than 1, thrombocytopenia, hypoalbuminemia, or symptoms and signs of portal hypertension should undergo the procedure [2].

## 3. Pathogenesis of NAFLD

Although the pathogenesis of NAFLD is not clearly understood, it is generally recognized that the causes of NAFLD are associated with components of metabolic syndrome such as insulin resistance, abdominal obesity, and a proinflammatory state [2, 16, 17]. Adipose tissue functions as a protective tissue by absorbing excess free fatty acids (FFA), thereby preventing overexposure to FFA in other organs, such as the liver, muscle, and pancreas. However, chronically elevated plasma FFA levels lasting from 24 to 72 hours can result in hypertrophy and hyperplasia of adipose tissue. The behavior of hypertrophic adipocytes is similar to that of macrophages and involves the activation of several inflammatory pathways and the secretion of adipokines, resulting in resistance of adipocytes to insulin. FFA-induced inflammatory responses include activation of macrophages [18], the shifting of Kupffer cells to the M1 phenotype which secretes proinflammatory cytokines such as tumor necrosis factor-*α*, inducible nitric oxide synthase, and interleukin-12 [19], and the activation of toll-like receptors [20]. At the same time, excess FFA can induce pancreatic beta cell dysfunction [21].

**Figure 2:**
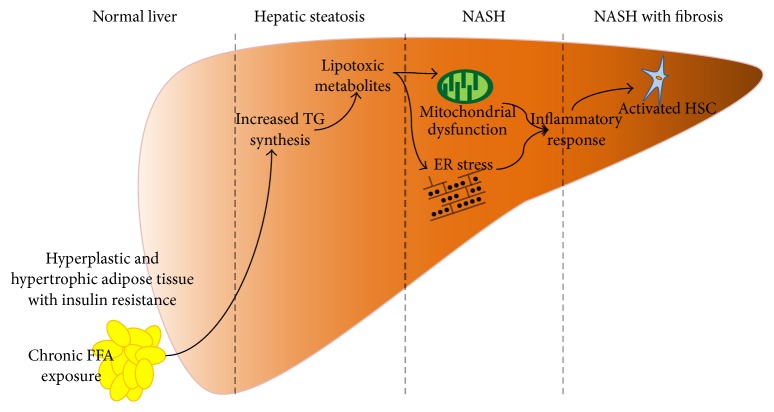
Pathogenesis of nonalcohol fatty liver disease. ER: endoplasmic reticulum; FFA: free fatty acid; HSC: hepatic stellate cell; NASH: nonalcohol steatohepatitis; TG: triglyceride.

Excessive TG accumulation in the liver due to increased levels of FFA in the blood stream is generally considered the first step in the development of NASH. In 1988, Day et al. established the two-hit hypothesis to explain the pathogenesis of NASH. According to that well-known hypothesis, the accumulation of hepatic fat sensitizes the liver to injury, resulting in an inflammatory response due to increased oxidative stress, endoplasmic reticulum stress, and increased levels of toxic metabolites caused by incomplete oxidation of fatty acids, such as diacylglycerols and ceramides (Figure 2) [22, 23].

## 4. Diagnosis of NAFLD and NASH

According to AGA and AASLD guidelines, the diagnostic criteria for NAFLD include imaging or histologic evidence of hepatic steatosis in patients without a history of significant alcohol consumption and without evidence of coexisting etiologies of chronic liver disease such as use of corticosteroids, HCV infection, and Wilson's disease [1]. Liver biopsy remains the gold standard for differentiating NASH from simple steatosis. Histologic evidence of NASH includes liver specimens with greater than 5% macro steatosis, the presence of lobular inflammation, and hepatocyte ballooning in acinar zone 3 [24]. Brunt et al. were the first to develop a semiquantitative method for grading necroinflammatory activity and staging fibrosis in patients with NASH [5]. According to their method, grading is based on the severity (mild, moderate, or severe) of steatosis, the degree of portal and intra-acinar chronic inflammation, and the presence of hepatocyte ballooning, and fibrosis staging is based on the patterns of fibrosis and degree of connective tissue deposition in zone 3 [5]. Since then, a number of refined histologic scoring systems have been developed, including the NAFLD activity score (NAS) [25] and the steatosis-activity-fibrosis (SAF) score [26]. The Asian Pacific Association for the Study of the Liver (APASL) recommends that the NAS scoring system be employed when evaluating NAFLD [27], and the AASLD reported that NAS is a useful tool for measuring changes in liver histology in clinical trials of patients with NAFLD [1]. Liver biopsy, however, is an invasive procedure, carries a risk of postprocedural infection, is contraindicated in certain patient groups, and has several limitations, including possible sampling bias. In addition, there is still no consensus about whether liver biopsy is required to confirm a diagnosis of NAFLD [1, 14, 27].

## 5. Noninvasive Assessment of Liver Fibrosis

Noninvasive alternatives to liver biopsy include measurement of biomarkers of fibrosis in serum, the use of diagnostic panels and scoring systems, and imaging studies. The most commonly measured biomarkers are aspartate aminotransferase (AST) and alanine aminotransferase (ALT). Although levels of those enzymes are elevated in 50% of patients with NAFL and in 80% of patients with NASH [15], studies have shown that tests for AST and ALT are not sensitive for predicting the severity of NAFLD [14].

A number of diagnostic panels and scoring systems have been proposed to assess the degree of fibrosis, such as the NAFLD fibrosis score (NFS) [28], the Fibrosis-4 (FIB-4) test [29], the BARD index [30], the AST-to-platelet ratio (APRI) [31], the FibroMeter [31], and the FibroTest [32]. The parameters, area under the receiver operating characteristic curves, cutoffs, sensitivities, specificities, positive predictive values, and negative predictive values of those fibrotic tests are shown in Table 1. Angulo et al. reported that an NFS score > 0.676 had an accuracy of 90% and a positive predictive value of 92% in distinguishing between patients with and those without advanced fibrosis [28]. A validation study in Hong Kong also reported that NFS is highly accurate in differentiating NAFLD patients with advanced fibrosis from those without advanced fibrosis [33]. The BARD index comprises three simple clinical parameters and the BARD score is the weighted sum of the three, namely, BMI ≥ 28 kg/m^2^ = 1 point, AST/ALT ratio ≥ 0.8 = 2 points, and diabetes = 1 point. Harrison et al. reported that a composite BARD score of 2–4 had an odds ratio of 17.3 for advanced fibrosis [30]. A study in Poland also demonstrated that a BARD score of 2–4 was associated with fibrosis stage F3 or F4 with an odds ratio of 17.3 [34]. Studies have shown that APRI at a cutoff of 1.0 and FIB-4 at a cutoff of <1.3 have low positive predictive values and high negative predictive values, indicating that they may be useful tools for differentiating between NAFLD patients with and those without advanced fibrosis [29, 31]. Sumida et al. found that FIB-4 had a negative predictive value of 98% at a cutoff of <1.45 but a positive predictive value of 53% at a cutoff of >3.25, indicating that implementation of the FIB-4 index would spare more than 50% of patients with NAFLD from undergoing liver biopsy [35]. A recent retrospective study showed that APRI, FIB-4, and the NAFLD fibrosis score were predictive of clinical outcomes and had similar prognostic performance to histologic fibrosis stage in patients with NASH [36].

Ultrasonography is the most commonly used imaging modality for diagnosing NAFLD. According to the Asian Pacific Association for the Study of the Liver (APASL), two or more of the following findings on ultrasound scans are diagnostic of NAFLD: (1) increased echogenicity of liver compared to kidney or spleen, (2) blurred vascularity, and (3) signal attenuation in deeper parts of the liver [27]. A prospective study revealed that ultrasound had a sensitivity of 100% and a specificity of 90% for diagnosing hepatic steatosis when hepatic fat content was greater than 20% but had low sensitivity (43%) and specificity (73%) for diagnosing microvesicular fat [37]. A meta-analysis of 49 studies revealed that ultrasonography had a pooled sensitivity of 84.8% and a specificity of 93.6% for detecting ≥20% steatosis and a diagnostic accuracy of 0.91 to 0.93 for detecting ≥10% steatosis [38]. However, the specificity for differentiating between steatosis and other pathologic findings, such as hepatitis or fibrosis, was only 79.2%. The meta-analysis also showed that ultrasound had a slightly better overall accuracy at detecting fatty liver than computed tomography or magnetic resonance imaging.

Other imaging modalities used for diagnosing NAFLD and for measuring the degree of fibrosis include transient elastography (TE) [39], ultrasound-based acoustic radiation force impulse (ARFI) elastography [40], and magnetic resonance elastography (MRE) [41]. TE uses a transducer probe to transmit an elastic shear wave with mild amplitude and low frequency, which propagates through the underlying tissue. An ultrasound probe is then used to follow the propagation of the produced shear wave and measure its velocity, which is directly related to hepatic stiffness [42]. Although TE is a promising tool to measure liver stiffness and estimate liver fibrosis, the modality is less effective in obese individuals (BMI > 28 kg/m^2^) because subcutaneous fat attenuates the elastic shear wave, resulting in reduced diagnostic performance [43]. In ARFI elastography, shear waves generated within tissues lead to transient tissue displacement, and the deformations can be ultrasonically tracked to estimate the degree of tissue stiffness [44]. In a head-to-head prospective clinical trial, Cui et al. showed that MRE had higher diagnostic accuracy than ARFI for diagnosing fibrosis in obese patients with NAFLD. However, they found that there were no differences in diagnosing fibrosis between the two modalities in nonobese patients with NAFLD [41].

## 6. Evaluation and Management of NAFLD

APASL guidelines for managing NAFLD include increasing levels of physical activity, dietary modification, screening for metabolic syndrome, and bariatric surgery for obese patients who fail to respond to lifestyle measures [27]. Although the management guidelines proposed by the European Association for the Study of the Liver (EASL) are similar to those proposed by the APASL, the EASL also recommends that physical exercise, reduction of sedentary lifestyle, weight loss, and dietary changes be assessed after a 6-month period [14]. Suzuki et al. showed that a 5% to 10% reduction in body weight was sufficient for normalization of AST and ALT levels [67]. In 2012, the AASLD reported that loss of 3% to 5% body weight can result in a reduction in the degree of steatosis and that a loss of up to 10% may be needed to reduce the degree of lobular inflammation and hepatocyte ballooning. In addition, the AASLD also recommended that vitamin E (800 IU/day) be administered as a first-line pharmacotherapy in nondiabetic patients with biopsy-proven NASH. Vitamin E, however, was not recommended for patients with diabetes mellitus, for those without histologic confirmation of NAFLD, or for patients with NAFLD-related liver cirrhosis [1]. All three international liver associations emphasized that metabolic diseases such as diabetes mellitus, hypertension, and dyslipidemia should be appropriately managed in patients with NAFLD (Table 2) [1, 14, 27].

## 7. Current Pharmacological Approaches in Western Medicine for Managing NASH

Current medical management of NASH includes vitamin E (800 IU/day), thiazolidinediones (TZDs, such as pioglitazone and rosiglitazone), pentoxifylline, and obeticholic acid. A recent meta-analysis revealed that vitamin E, pentoxifylline, and obeticholic acid result in a reduction in degree of fibrosis and that vitamin E, TZDs, and obeticholic acid result in a reduction in ballooning degeneration in patients with NASH. The therapeutic effects of those drugs in patients with NAFLD did not differ significantly [46]. However, long-term intake of vitamin E (400 IU/day for more than 6 years) has been shown to increase the risk of prostate cancer [47]. In addition, a large-scale randomized controlled trial revealed that long-term intake of pioglitazone increased the risk of bladder cancer in patients with type 2 diabetes and evidence of macrovascular disease (PROactive study) [49]. Although concerns have been raised about the validity of the results of the PROactive study [68, 69], a recent systematic review showed that pioglitazone may be associated with the development of other types of cancer [70]. Rosiglitazone has been reported to increase the risk of myocardial infarction and congestive heart failure in patients with type 2 diabetes [50]. In the FLINT study, obeticholic acid was found to increase low-density lipoprotein (LDL) levels in patients with NASH [51] (Table 3). Recently, liraglutide, a glucagon-like peptide 1, was shown in two small clinical trials to be a safe and well-tolerated drug that led to the histological resolution of nonalcoholic steatohepatitis [71, 72]; however, larger scale clinical trials with longer follow-up periods are needed to further evaluate the safety and efficacy of this drug.

## 8. Recent Studies of TCM for Treatment of NAFLD

### 8.1. Cell Studies

The effects of a number of traditional Chinese herbs on metabolic parameters associated with NAFLD have been studied in vitro. For example, Wang et al. reported that treatment of L02 hepatocytes that had been grown in medium containing fat emulsion and a high concentration of fetal bovine serum with an extract of radix of* Polygoni multiflori* Moldenke (RPM) as well as its active components emodin, physcion, and 2,3,5,4′-tetrahydroxystilbene-2-O-*β*-D-glucoside resulted in a decrease in TG, TC, and low-density lipoprotein (LDL) levels [52]. Kang and Koppula reported that ethanol extract of* Houttuynia cordata* Thunb. (HC) attenuated lipid accumulation and downregulated the expression of fatty acid synthase (FAS), sterol regulatory element-binding protein- (SREBP-) 1c, and glycerol 3-phosphate acyltransferases (GPATs) in HepG2 cells that had been exposed to 25 mM of glucose for 24 hours [53]. They, however, did not mention which of the four GPAT isoforms was affected by HC treatment. Lee et al. showed that methanol extract of* Ixeris dentata* (IXD) inhibited hepatic accumulation of TG and TC by regulating endoplasmic reticulum (ER) stress in HepG2 cells in the presence of palmitate [54]. Kang et al. found that curcumin and puerarin suppressed lipid accumulation, resulted in a reduction in expression of SREBP-1 and FAS, and led to an increase in peroxisome proliferator activated receptor- (PPAR-) *α* expression in HepG2 cells treated with oleic acid [55, 56].

**Figure 3:**
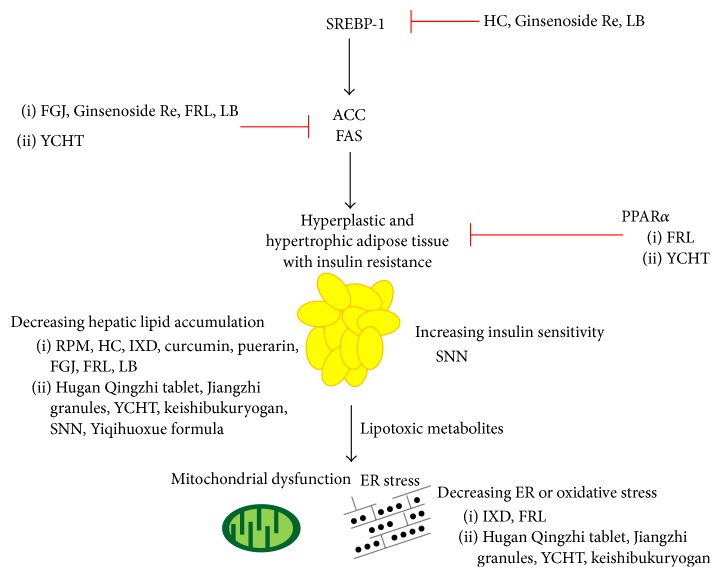
Mechanisms of TCM in the pathogenesis of NAFLD. ACC: acetyl-CoA carboxylase; ER: endoplasmic reticulum; FAS: fatty acid synthase; FGJ: fruit of* Gardenia jasminoides* J. Ellis; HC:* Houttuynia cordata *Thunb.; IXD:* Ixeris dentata*; LB:* Lycium barbarum* L.; PPAR: peroxisome proliferator activated receptor; FRL: fruit of* Rosa laevigata* Michx.; RPM: radix of* Polygoni multiflori* Moldenke; SNN: Salvia-Nelumbinis naturalis; SREBP: sterol regulatory element binding proteins; YCHT: Yin-Chen-Hao-Tang.

Hugan Qingzhi tablet, a lipid-lowering TCM formula comprising* Alisma plantago-aquatica* L. (APA),* Crataegus pinnatifida* Bunge (CP),* Typha orientalis* C. Presl, leaf of* Nelumbo nucifera* Gaertn. (NN), and radix of* Panax notoginseng* F. H. Chen, has been demonstrated to reduce lipid accumulation as well as AST, ALT, lactate dehydrogenase, and malondialdehyde (MDA) levels and to increase the generation of superoxide dismutase (SOD) and glutathione (GSH) in L02 and HepG2 cells [57]. Protopanaxadiol, tanshinone IIA, and emodin, three of the five components of the TCM formula Salvia-Nelumbinis naturalis (SNN, initially referred to as Jiangzhi granules containing protopanaxadiol, tanshinone IIA, emodin, chlorogenic acid, and nuciferine), were found to ameliorate lipid accumulation and protopanaxadiol, but not tanshinone IIA or emodin, was shown to decrease ROS generation in HepG2 cells that had been exposed to FFA for 24 hours [58] (Figure 3 and Table 4).

### 8.2. Animal Studies

The effects of a number of traditional Chinese herbs on metabolic parameters associated with NAFLD have also been studied in vivo. For example, water extract of fruit of* Gardenia jasminoides* J. Ellis (FGJ) administered via gastric gavage was shown to have a protective effect against lipid accumulation and inflammatory injury in rats that had been fed a high fat diet (HFD) [59]. Quan et al. reported that Ginsenoside Re had antidiabetic and antihyperlipidemic activities via induction of the orphan nuclear receptor small heterodimer partner (SHP) and suppression of SREBP-1c and its target genes* FAS* and* SCD-1*, which code for fatty acid synthase and stearoyl-CoA desaturase-1, in HepG2 cells and in HFD-fed mice [60]. Total saponins from fruit of* Rosa laevigata* Michx. were demonstrated to attenuate hepatic steatosis and lipotoxic oxidation and inflammation, result in a reduction in body weight and AST, ALT, TC, TG, FFA, LDL, blood glucose, insulin, and MDA levels, and lead to increased high-density lipoprotein and GSH levels in HFD-fed rats [61]. Administration of a water/ethanol extract of* Lycium barbarum* L. polysaccharides was shown to protect against the development of hepatic steatosis, improve lipid metabolic profiles, and suppress the expression of FAS and SREBP-1c in HFD-fed mice [62]. In a review of recent studies on the effects of Semen of* Phyllolobium chinense* Fisch. (SPC) on metabolic parameters associated with NAFLD, Ng et al. found that active contents of SPC including fatty acids, amino acids, polysaccharides, flavonoids, and triterpene glycosides had antihypertensive, antidiabetic, anticancer, antioxidant, and antifibrotic effects and that administration of crude extract of SPC as well as administration of total flavonoids and triterpene glycosides in SPC had lipid-lowering and liver protection effects in rats that had been fed a high lipid diet [73].

Lee et al. found that the TCM herbal formula Yin-Chen-Hao-Tang (containing three medicinal herbs:* Artemisia capillaries* Thunb. (AC), FGJ, and* Rheum officinale* Baill. (RO)) administered to HFD-fed rodents for a period of 15 weeks resulted in an increase in level of adiponectin, an increase in level of circulating endothelial progenitor cells, the upregulation of PPAR-*γ*, and elevated GSH level in hepatic tissue [63]. Fujimoto et al. found that oral administration of Kampo formula keishibukuryogan (KBG) (components: cortex of* Cinnamomum cassia* J. Presl, radix of* Paeonia lactiflora* Pall., semen of* Prunus persica* Batsch,* Poria cocos* Wolf., and cortex of* Paeonia suffruticosa* Andrews) for twelve weeks resulted in significant reductions in hepatic TG, fibrosis, and oxidative stress in a rabbit model of NASH [64]. In addition, Zhang et al. showed that SNN alleviated hepatosteatosis and improved lipid profiles in Wistar rats that had been exposed to a high caloric diet [65]. Chen et al. demonstrated in a murine model of NAFLD that administration of Yiqihuoxue formula (components: FGJ, radix of* Sedum kirilowii* Regel, rhizoma of* Curcuma longa* L., and fructus of* Ligustri lucidi* W. T. Aiton) for five weeks resulted in improved liver function and a normolipoproteinemia profile via decreasing expression of TNF-*α* [66] (Figure 3 and Table 4).

### 8.3. Clinical Studies

In a multicenter clinical trial, short-term administration of Danning Pian, a TCM herbal formula comprising RO,* Reynoutria japonica* Houtt. (RJ), immaturus fructus of* Citrus* ×* aurantium* L. (IFCA), and pericarpium of* Citrus* ×* aurantium* L. (PCA), was shown to improve clinical symptoms, reduce ALT and TC levels, and result in a mild reduction in degree of steatosis in patients with NAFLD. The trial, however, did not report the results of patients in the control group who received ursodeoxycholic acid (UDCA) [74]. In a clinical trial comparing the effectiveness of QuYuHuaTanTongLuo (QYHTTL) decoction (components: radix of* Bupleurum falcatum* L., radix of* Scutellaria baicalensis* Georgi,* Pinellia ternata* Makino, radix of* Codonopsis pilosula* Nannf., radix of* Glycyrrhiza glabra* L., fructus of* Ziziphus jujuba* Mill., RJ, radix of* Gynochthodes officinalis* Razafim. & B. Bremer, and* Oldenlandia diffusa* Roxb.) with that of UDCA in patients with NASH, Zhang et al. found that patients who received the decoction for six months had significantly lower levels of hepatic aminotransferases and significantly better lipid profiles than patients who received UDCA. The researchers also provided evidence that the effect of QYHTTL is due, at least in part, to its anti-inflammatory and antioxidant properties [75]. In a retrospective observational study, Fujimoto et al. reported that patients with NAFLD who received KBG for 8–12 weeks showed significantly lower AST, ALT, and TC levels after treatment [76] (Figure 3 and Table 4).

However, a Cochrane review in 2013 showed that although some TCM formulas, such as Jiangzhi Ligan decoction (composition:* Sargassum Pallidum* Turn.,* Salvia miltiorrhiza* Bunge (SM),* Cassia obtusifolia* H. S. Irwin & Barneby (CO), APA, NN,* Curcuma wenyujin* Salisb.,* Curcuma longa* L. (CL),* Hirudo nipponica*, and* Bupleurum chinense* DC. (BC)), Chaihu Shugan powder (composition: BC, PCA, IFCA,* Cyperus rotundus* L.,* Melia azedarach* L., CP, SM,* Paeonia albiflora* Pall.,* Rheum palmatum* L., APA,* Polygonum multiflorum* Moldenke, AC,* Paeonia* ×* suffruticosa* Andrews, FGJ, CL,* Curcuma aromatica* Salisb.,* Trionyx sinensis*,* Cornus officinalis* Seibold & Zucc.,* Ligustrum lucidum* W. T. Aiton, and* Rehmannia glutinosa* DC. (RG)), and Qingzhifugan decoction (composition: RG, SM,* Poria cocos* Wolf.,* Atractylodes macrocephala* Koidz.,* Sophora flavescens* Aiton, CP, CO, and RPM) have positive effects on AST, ALT, and ultrasonographic findings in NAFLD patients, meta-analysis was not performed and no conclusions on the effectiveness of those TCM formulas could be reached because of the heterogeneity of the TCM trials included in the review, including different TCM formulas, the limited number of participants, and different outcome measures [77].

## 9. Opportunities and Challenges for TCM as Treatment for NAFLD

Lifestyle modifications such as weight loss, dietary changes, and physical exercise are strongly recommended to decrease the risk of metabolic disease in patients with NAFLD [1, 14, 27]; however, the majority of people find it difficult to achieve public health guidelines, such as attaining a minimum of 150 minutes per week of moderately vigorous physical activity [78, 79]. In addition, although a few pharmaceutical agents have been approved for NAFLD, many are associated with potential side effects or are only indicated in certain patients. For example, vitamin E has been shown to increase the risk for prostate cancer [47] and can only be used in nondiabetic patients with biopsy-proven NASH [1]. Pioglitazone and rosiglitazone have been demonstrated to increase the risk for bladder cancer [49] and myocardial infarction [50], and obeticholic acid has been shown to result in elevated LDL [51].

Cellular and animal studies have demonstrated that some TCM herbs and formulas can play a role in blocking the pathogenic pathways of NAFLD. TCM drugs have been shown to reduce AST and ALT levels, improve lipid profiles, suppress the activity of lipogenic enzymes, such as SREBP-1c, GPAT, and FAS [53, 55, 56, 60–62], improve hepatic insulin sensitivity [65], have anti-inflammatory effects [59], increase adiponectin levels, upregulate PPAR-*γ* [57, 63], and have antioxidant properties [58, 63, 80]. Table 4 provides a summary of the metabolic effects of a number of TCM herbs and formulas reported in various cellular and animal studies.

Clinical trials are fundamental for evaluating the efficacy and safety of drugs in humans [81]. Study designs of trials are important and, unfortunately, many TCM clinical studies used insensitive methods to evaluate the severity of NAFLD, such as measuring AST and ALT levels [14]. Future TCM trials should use noninvasive serologic methods or liver biopsy to evaluate the severity of NAFLD [36]. In addition, many TCM studies on NAFLD suffer from selection, performance, detection, and reporting biases and lack homogenous clinical data [77]. For example, in a large meta-analysis of the effects of herbal medicines on NAFLD-related biochemical parameters, Liu et al. found no conclusive evidence to support the effectiveness of herbal agents because of the high risk of bias in the trials and because of the heterogeneity of the data [77]. In addition, reproducible methods are needed to evaluate the effectiveness of specific TCM physical examinations, such as tongue inspection [82] and pulse examination [83, 84] for the detection of NAFLD.

Therefore, although there is evidence that certain TCM herbs and formulas have an effect on NAFLD, well-designed TCM clinical trials are warranted to better understand the efficacy and safety of TCM drugs as treatment for nonalcoholic fatty liver disease.

## Figures and Tables

**Table 1:** Fibrotic tests based on biochemical variables.

Test	Parameters	AUROC	Cutoffs	Sensitivity	Specificity	PPV	NPV
NAFLD fibrosis score [28]	IFG/DM, AST/ALT, age, BMI, PLT, albumin	0.82–0.88	<−1.455	77%	71%	52%	88%
			>0.676	43%	96%	82%	80%

FIB-4 [29, 45]	ALT, AST, PLT, age	0.80	<1.3	74%	71%	43%	90%
			≥2.67	33%	98%	80%	83%

BARD [30]	BMI, AST/ALT, DM	0.81	2	—	—	43%	96%

APRI [29, 31]	AST, PLT	0.82	1.0	67%	81%	31%	95%

FibroMeter [31]	Glucose, ALT, AST, weight, age, PLT, ferritin	0.93–0.94		78%	96%	88%	92%

FibroTest [32]	Age, sex, bilirubin, GGT, apolipoprotein A1, haptoglobin, *α*2-macroglobulin	0.81–0.92	>0.3	92%	71%	33%	98%
			>0.7	25%		60%	89%

ALT: alanine aminotransferase; APRI: AST-to-platelet ratio; AST: aspartate aminotransferase; AUROC: area under receiver operating characteristic curve; BMI: body mass index; DM: diabetes mellitus; FIB-4: Fibrosis-4 test; GGT: *γ*-glutamyl transferase; IFG: impaired fasting glucose; NAFLD: nonalcohol fatty liver disease; NPV: negative predictive value; PLT: platelets; PPV: positive predictive value.

**Table 2:** AASLD, EASL, and APASL guidelines for diagnosing and managing NAFLD.

Association	AASLD [1]	EASL [14]	APASL [27]
Evaluate liver fibrosis	NAFLD fibrosis score	Combination of serum markers and imaging method (elastometry)	None

Indication for liver biopsy	(i) NAFLD patients at increased risk of steatohepatitis and advanced fibrosis (ii) Metabolic syndrome and the NAFLD fibrosis score may be used to identify patients at risk	(i) Noninvasive methods to evaluate fibrosis in patients with insulin resistance, increased ALT, or steatosis (ii) Liver biopsy in advance-fibrotic patients with evidence of noninvasive methods or insufficient data to exclude advanced fibrosis	(i) Diagnostic uncertainty (ii) Risk of advanced hepatic fibrosis without evidence of cirrhosis (iii) Clinical trial (iv) Subject to laparoscopy for another purpose, such as cholecystectomy, gastric banding

Lifestyle intervention	(i) 3–5% weight loss to improve steatosis, a greater weight loss (>7% to 10%) to improve necroinflammation (ii) Exercise	(i) Physical exercise, 5–10% weight loss (aim: 7%), dietary changes (ii) Reassess after 6 months (iii) Avoid fructose corn syrup and industrial *trans*-fats	(i) Diet, increasing physical activity (aerobic exercise), weight reduction (ii) Bariatric surgery or gastric ballooning should be considered in patients without response to lifestyle measures

Medications	(i) Pioglitazone in patients with biopsy-proven NASH; long-term safety and efficacy were not established (ii) Vitamin E (800 IU/day) for nondiabetic adult with biopsy-proven NASH (iii) Statins can be used to treat dyslipidemia in patients with NAFLD (iv) UDCA and omega-3 fatty acids were not recommended	(i) Correct concurrent metabolic disorders (ii) Suggest further therapeutic trials	Statin for NAFLD and usual indications

Other	Assess for metabolic risk factors and alternative etiologies for NASH		Monitor abdominal girth, body weight, fasting blood glucose, serum lipid, blood pressure and screening cancers increased by metabolic syndrome

AASLD: American Association for the Study of Liver Disease; ALT: alanine aminotransferase; APASL: the Asian Pacific Association for the Study of the Liver; EASL: European Association for the Study of the Liver; NAFLD: nonalcoholic fatty liver disease; NASH: nonalcohol steatohepatitis; UDCA: ursodeoxycholic acid.

**Table 3:** Pharmacological interventions for NAFLD.

Medication	Indications	Contraindications	Limitations	Side effects
Vitamin E [46]	Nondiabetic patients with NASH	Prostate cancer	Increasing risk of prostate cancer and hemorrhagic stroke [47]	(i) Increased risk of hemorrhagic stroke [47]
				(ii) Increased mortality above recommended daily allowances [48]

TZD [46]	(i) Diabetic patients with NAFLD	Symptomatic heart failure	(i) Pio: increasing risk of bladder cancer [49]	Weight gain, bone loss, GI upset, fatigue, lower extremity edema
	(ii) NASH		(ii) Rosi: myocardial infarction [50]	

OCA [46, 51]	NASH	Not commercialized	(i) Not available in clinical practice	Pruritus, elevated LDL
			(ii) Long-term safety is unknown	

GI: gastrointestinal; LDL: low-density lipoprotein; NAFLD: nonalcohol fatty liver disease; NASH: nonalcohol steatohepatitis; OCA: obeticholic acid; Pio: pioglitazone; Rosi: rosiglitazone; TZD: thiazolidinedione.

**Table 4:** Cellular and animal studies of TCM for NAFLD.

Agent	Testing subjects	Function of treatment	Reference
Radix of *Polygoni multiflori* Moldenke, emodin, physcion, and 2,3,5,4′-tetrahydroxystilbene-2-O-*β*-D-glucoside	L02 cell line with fat emulsion medium and high conc. of FBS	Decreasing cellular TG, TC, LDL content	[52]

*Houttuynia cordata *Thunb.	HepG2 cell line with glucose	Decreasing hepatic glucose, TG, TC accumulation Inhibiting SREBP-1c, GPAT	[53]

*Ixeris dentata*	HepG2 cell line with palmitate	Decreasing hepatic lipid accumulation, TG, TC Inhibiting palmitate-induced ER stress	[54]

Curcumin and puerarin	HepG2 cell line with oleic acid	Decreasing lipid accumulation, TG, TC Inhibiting SREBP-1, FAS Increase PPAR-*α*	[55, 56]

Hugan Qingzhi tablet	L02 and HepG2 cell line with FFAs	Decreasing lipid accumulation, TG, LDH, AST, ALT, MDA Increasing GSH, SOD Inhibiting SREBP-1, increase PPAR-*α*, CPT-1, ACOX1	[57]

Protopanaxadiol, tanshinone IIA, and emodin (active component of Jiangzhi granules)	HepG2 cell line with FFAs	Decreasing lipid accumulation, ROS	[58]

Fruit of* Gardenia jasminoides *J. Ellis	Sprague Dawley rats with high fat diet	Decreasing hepatic TG, FFA, AST, ALT Inhibiting FAS, malonyl-CoA, ACC	[59]

Ginsenoside Re	HepG2 cell line C57BL/6J mice with high fat diet	Decreasing glucose production, TG, FFA, insulin, HOMA-IR Inhibiting SREBP-1c, FAS, GPAT	[60]

Fruit of *Rosa laevigata* Michx.	Wistar rats with high fat diet	Decreasing hepatic lipid accumulation, FFA, TG, TC, LDL, AST, ALT Increasing GSH Inhibiting SREBP-1c, FAS, ACC, GPAT Increase PPAR-*α*, CPT-1	[61]

*Lycium barbarum *L.	HepG2 cell line with palmitate C57BL/6J mice with high fat diet	Decreasing mice weight, hepatic lipid accumulation, blood glucose, TG, TC, LDL, AST, ALT, FFA Inhibiting SREBP-1c, FAS, ACC Increasing CPT-1	[62]

Yin-Chen-Hao-Tang	Hamsters with high fat diet	Decreasing balloon hepatocytes, TG, FFA, TNF-*α*; increasing adiponectin, GSH Inhibiting FAS; increasing PPAR-*α*	[63]

keishibukuryogan	White rabbit	Decreasing hepatic lipid accumulation, plasma and liver oxidative stress, liver fibrosis	[64]

Salvia-Nelumbinis naturalis (Jiangzhi granules)	HepG2 cell line with FFAs Wistar rat with high caloric diet	Decreasing hepatic lipid accumulation, TG, hepatic glycogen, TG, LDL, FFA Improving insulin sensitivity	[65]

Yiqihuoxue formula	Sprague Dawley rats with high fat diet	Decreasing rat weight, hepatic lipid accumulation, TG, ALT	[66]

ACC: Acetyl-CoA carboxylase; ACOX1: acyl-CoA oxidase 1; ALT: alanine aminotransferase; AST: aspartate aminotransferase; CPT-1: carnitine palmitoyltransferase 1; ER: endoplasmic reticulum; FAS: fatty acid synthase; FBS: fetal bovine serum; FFAs: free fatty acids; GSH: glutathione; GPAT: glycerol 3-phosphate acyltransferases; HOMA-IR: homeostasis model assessment of insulin resistance; LDH: lactate dehydrogenase; LDL: low-density lipoprotein; MDA: malondialdehyde; PPAR: peroxisome proliferator activated receptor; NAFLD: nonalcohol fatty liver disease; ROS: reactive oxygen species; SOD: superoxide dismutase; SREBP: sterol regulatory element-binding proteins; TCM: traditional Chinese medicine; TG: triglyceride; TC: total cholesterol.
